# Bioreactor With Electrically Deformable Curved Membranes for Mechanical Stimulation of Cell Cultures

**DOI:** 10.3389/fbioe.2020.00022

**Published:** 2020-01-28

**Authors:** Joana Costa, Michele Ghilardi, Virginia Mamone, Vincenzo Ferrari, James J. C. Busfield, Arti Ahluwalia, Federico Carpi

**Affiliations:** ^1^Research Center “E. Piaggio”, University of Pisa, Pisa, Italy; ^2^Department of Information Engineering, University of Pisa, Pisa, Italy; ^3^School of Engineering and Materials Science, Queen Mary University of London, London, United Kingdom; ^4^Materials Research Institute, Queen Mary University of London, London, United Kingdom; ^5^Department of Information Engineering, EndoCAS Center for Computer Assisted Surgery, University of Pisa, Pisa, Italy; ^6^Department of Industrial Engineering, University of Florence, Florence, Italy

**Keywords:** actuator, bioreactor, cell, dielectric elastomer, electroactive polymer, mechanical stimulation, membrane, stretch

## Abstract

Physiologically relevant *in vitro* models of stretchable biological tissues, such as muscle, lung, cardiac and gastro-intestinal tissues, should mimic the mechanical cues which cells are exposed to in their dynamic microenvironment *in vivo*. In particular, in order to mimic the mechanical stimulation of tissues in a physiologically relevant manner, cell stretching is often desirable on surfaces with dynamically controllable curvature. Here, we present a device that can deform cell culture membranes without the current need for external pneumatic/fluidic or electrical motors, which typically make the systems bulky and difficult to operate. We describe a modular device that uses elastomeric membranes, which can intrinsically be deformed by electrical means, producing a dynamically tuneable curvature. This approach leads to compact, self-contained, lightweight and versatile bioreactors, not requiring any additional mechanical equipment. This was obtained via a special type of dielectric elastomer actuator. The structure, operation and performance of early prototypes are described, showing preliminary evidence on their ability to induce changes on the spatial arrangement of the cytoskeleton of fibroblasts dynamically stretched for 8 h.

## Introduction

All biological tissues are subjected to internal mechanical forces that arise from interstitial flows and cellular motions. These forces can redistribute effector molecules that are secreted by cells, resulting in the coupling of chemical and mechanical signaling. This phenomenon, known as mechanotransduction, is a major research interest in the fields of regenerative medicine and tissue engineering (Griffith and Swartz, 2006).

Static or cyclic and axial or biaxial strains applied to monolayers of cells, cultured on deformable membranes or 3D scaffolds (Elsaadany et al., 2017), have been used for decades (Leung et al., 1977; Meikle et al., 1979) to show that mechanical stretch can induce cell proliferation, increase tissue organization and enhance mechanical properties of cultured tissues. At present, most of the commercially available devices for cell stretching *in vitro* are actuated by pneumatic systems, such as those from Flexcell^®^ (Flexcell International, 2019), or mechanical motors, such as those from Strex^®^ (Strex Inc, 2019). They require external driving units (vacuum pumps or motors), which make the systems bulky, complex to operate, acoustically noisy and generally capable of low throughput (Brown, 2000).

Other systems are designed to mechanically stimulate cells at the microscopic scale, so as to study the response of few or even single cells, with so-called organ-on-a-chip devices (Clark et al., 2000; Akbari and Shea, 2012a, b; Kim et al., 2012; Pavesi et al., 2015). They are based on microfluidic systems, which advantageously host cells in highly miniaturized chambers, although they are still limited by the need for much bulkier external fluidic components.

In order to obtain more compact and easier-to-use systems, the potential usage of smart materials (not requiring external pumps/motors) is of growing interest. In particular, within the family of electromechanically active polymers (Carpi, 2016), dielectric elastomer actuators (DEAs) at present can in general offer large strains (10–100%) and relatively high stresses (up to 1 MPa) in response electrical stimuli, with simple structure, compact size, light weight, and low power consumption (Pelrine et al., 2000; Carpi et al., 2008). Due to these attractive properties, their potential also for the mechano-stimulation of cells has recently been explored (Akbari and Shea, 2012a, b; Cei et al., 2016; Poulin et al., 2016, 2018; Imboden et al., 2019). However, so far, they have been used for cell stretching of planar (uni- or bi-directional, or radial) and uniform kind. As a difference, tissues *in vivo* mostly undergo stretch fields that are non-planar, anisotropic, and inhomogeneous (Balestrini et al., 2010). So, the greatest potential of these smart materials to mimic physiologically relevant conditions remains at present mostly unexplored.

Here, we target a mechanical stimulation of cellular cultures on surfaces having a dynamically controllable curvature. As an alternative to conventional hydraulic/fluidic systems that can achieve an analogous effect, we present a DEA-based modular device made of elastomeric membranes that are deformable electrically (i.e., their curvature can be dynamically tuned by purely electrical means), without any fluidic system. This new approach is shown to lead to compact, self-contained and noise-free bioreactors that do not require any additional external mechanical equipment, as detailed below.

## Methods

**FIGURE 1:**
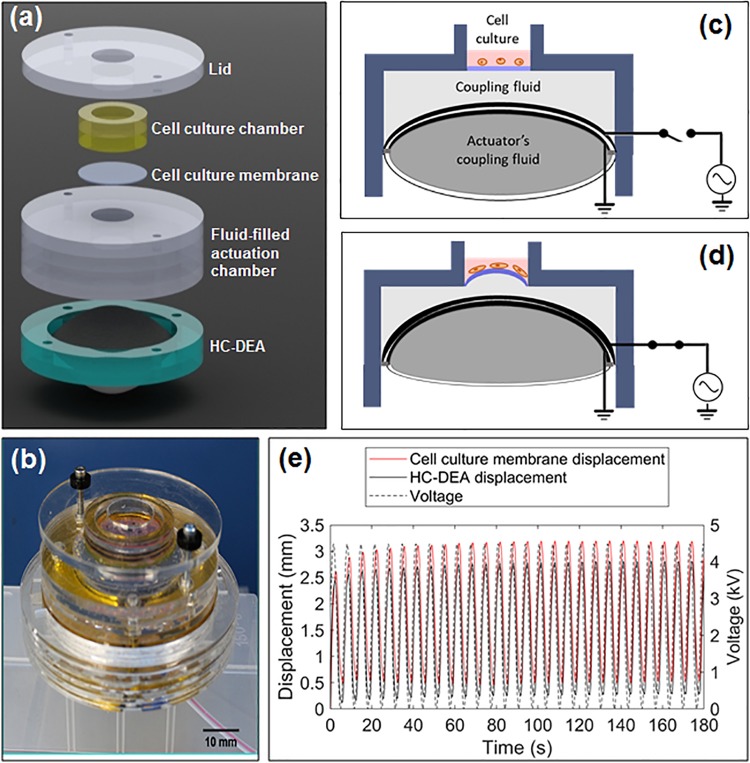
Proposed bioreactor with electrically deformable curved membrane: **(a)** exploded view of the structure; **(b)** picture of an assembled prototype; **(c,d)** schematic representation of the principle of operation; **(e)** examples of displacement signals (culture membrane and actuator) in response to a cyclic voltage.

The structure of the proposed bioreactor and its principle of operation are presented in Figures 1a,b. An elastomeric cell culture membrane is arranged on top of a fluid-filled chamber that contains also a soft actuator. With respect to conventional hydraulic/fluidic systems, the fluid here has a different function, as explained below. The cell culture membrane seals the chamber and is in contact with the fluid. When the actuator is off (no applied voltage), the membrane surface can either be flat or, if needed, have an initial pre-curvature (obtained by increasing the fluid volume). In response to an applied voltage, the actuator is able to vary the cell culture membrane’s curvature, according to a variable displacement of the fluid confined underneath, as detailed below.

The soft actuator consists of a special type of DEA, known as hydrostatically coupled dielectric elastomer actuator (HC-DEA) (Carpi et al., 2010). It is obtained by combining two dielectric elastomer circular films together and confining a coupling fluid between them, so as to obtain a bubble-like shape. One membrane (the one at the top in Figure 1c) is made active by sandwiching it between two compliant electrodes connected to a voltage source, while the other membrane (the one at the bottom in Figure 1c) is passive. When a voltage is applied to the active membrane, the bubble-like structure of the actuator deforms upward (Figure 1d). This is due to an electrically induced reduction of the active membrane’s thickness and an increase in its area, while the passive membrane buckles in the same direction as a result of the fluid-mediated coupling (Carpi et al., 2010).

This electrically induced deformation of the actuator is then used to displace the second coupling fluid confined above it (Figure 1d), so that the cell culture membrane can assume a controllable curvature, depending on the magnitude of the applied voltage. It is worth noting that voltages of opposite polarity and same amplitude cause the same curvature, as the actuation pressure generated by the HC-DEA is dependent on the square of the applied voltage (Carpi et al., 2010), as for any other DEA (Carpi et al., 2008). It is also useful to remark that the actuator’s structure could easily be modified by making both of its membranes active (i.e., providing both of them with electrodes) and independently controllable, so as to make it able to buckle in both directions: this would enable bi-directional displacements of the cell culture membrane, in order to obtain both tuneable convexities and tuneable concavities.

According to this principle of operation, a voltage (either static or dynamic) applied to the soft actuator can be used to generate a deformation of the cell culture membrane, stretching any adhered cells both circumferentially and radially. The Supplementary Video S1 shows a prototype in action.

Prototypes of this device were manufactured as follows. For the actuator’s active and passive membranes, a 1 mm-thick acrylic-based elastomeric film (VHB^TM^ 4910, 3M, United States) was biaxially pre-strained by 250% (i.e., 3.5 times pre-stretched), reaching an estimated thickness of about 82 μm, and fixed to a plastic circular frame with an internal diameter of 50 mm. The active membrane’s compliant electrodes were manufactured using a custom-made conductive ink, consisting of a dispersion of 9 wt% carbon black (Black Pearls 2000, Cabot, United States) in a silicone pre-polymer (MED4901, NuSil, United States) dissolved in isooctane (Sigma Aldrich) with a 1:1 volume ratio. The reagents were mixed using a planetary mixer (THINKY ARE-250, Intertronics, United Kingdom) and the resulting ink was sprayed with an airbrush onto the active membrane’s surfaces, where the silicone matrix was cured, obtaining elastomeric electrodes.

The actuator was then assembled by pulling the passive membrane with a custom-build vacuum chamber, filling the resulting cavity with 15 ml of a fluid silicone pre-polymer (Transil 40, Mouldlife, United Kingdom) and finally closing the cavity with the active membrane. The adhesive properties of the VHB elastomeric film allowed for sufficient bonding. The two membranes and the fluid encapsulated between them formed a bubble-like structure, with the fluid acting as a hydrostatic coupling medium between the membranes.

The cell culture membrane was manufactured by film casting with a silicone elastomer (Silbione LSR 4305, BlueStar Silicones, Norway). It had a thickness of about 75 μm and a diameter of 16.5 mm. A fluid-mediated hydrostatic coupling was also established between the actuator and the cell culture membrane, so as to transfer motion from the former to the latter. For simplicity, the adopted fluid was the same silicone pre-polymer used inside the actuator. The cell culture chamber was fixed to the rest of the structure with screws, so as to simplify the interchangeability among different types of chambers, enabling system modularity.

The diameter of the cell culture membrane was smaller than that of the actuator. Therefore, the fluid mediated coupling between surfaces of different area resulted in a larger vertical displacement in the cell culture membrane with respect to that of the actuator. This effect (maximized by an incompressible fluid) is evident from the sample signals shown in Figure 1e. Hence, changing the size of the cell culture membrane could be used to change the maximum achievable curvature at the maximum voltage, for any given actuator size. Moreover, for any given size of the cell culture membrane, the achievable curvature can always be modulated via the applied voltage.

A preliminary characterization of prototypes of this new device is presented below.

## Results and Discussion

### Frequency Response Over Time

**FIGURE 2:**
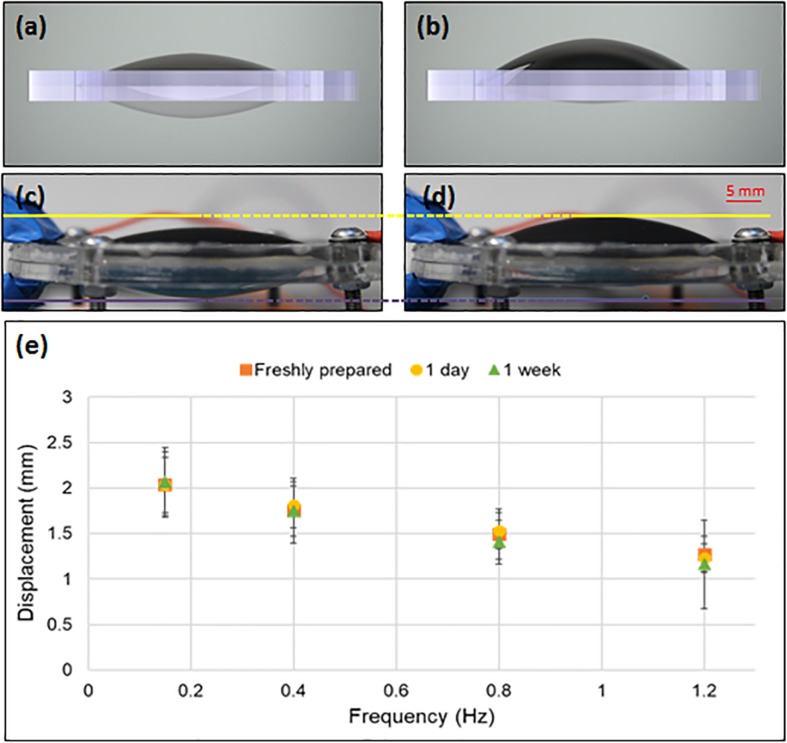
Operation of the HC-DEA and its frequency response. The drawings **(a,b)** and pictures **(c,d)** of the device show it at electrical rest **(a,c)** and with an applied voltage of 4.5 kV **(b,d)**. Panel **(e)** presents the frequency response to 4.5 kV sinusoidal waves in terms of maximum displacement of the central (highest) point of the active membrane, as measured at different times after fabrication: 0, 1, and 7 days. Error bars represent the standard deviation among three samples.

The HC-DEA dynamic performance was tested by measuring, with a laser-based displacement transducer (optoNCDT 1800, Micro-Epsilon, Germany), the active membrane’s maximum displacement (Figures 2a–d) in response to unipolar sinusoidal voltages.

As these tests were aimed at assessing the frequency response and how it changes over time, the amplitude of the voltage signals was fixed (4.5 kV, according to the thickness of the active membrane) and the frequency was varied within the 0.15–1.2 Hz range, corresponding to characteristic frequencies of intestinal, lung and cardiac tissue motions (Saul, 1990; Taylor and Eckberg, 1996; Han et al., 1998). The signals were obtained from a custom-made generator based on a miniature high voltage multiplier (Q50, EMCO High Voltage Corporation, United States). For each tested frequency, the voltage signal was applied for 3 min, followed by 1 min of rest. The experiments were performed right after fabrication and then repeated after 1 and 7 days.

Figure 2e presents the results, showing that, as expected, there was a notable decrease of the achievable displacement as the frequency increased. This can be ascribed to the viscous components of the constitutive elastomeric membrane (Carpi et al., 2008). Nevertheless, for each frequency, the average displacement was found to be stable over time, at least over the 7 days investigated (Figure 2e). This makes the technology potentially usable for continuous cell stretching over several days.

### Vertical Displacements and Radial and Circumferential Strains

In order to investigate the deformation occurring in different regions of the cell culture membrane and thus assess the mechanical stimuli imposed to cells adhered over its surface, the following tests were performed.

The bioreactor was driven with a sinusoidal voltage of 4.5 kV at a frequency of 0.15 Hz and the bi-dimensional distribution of the vertical displacement (displacement field map) and mono-dimensional (radial) distribution of both the radial and circumferential strains were estimated.

**FIGURE 3:**
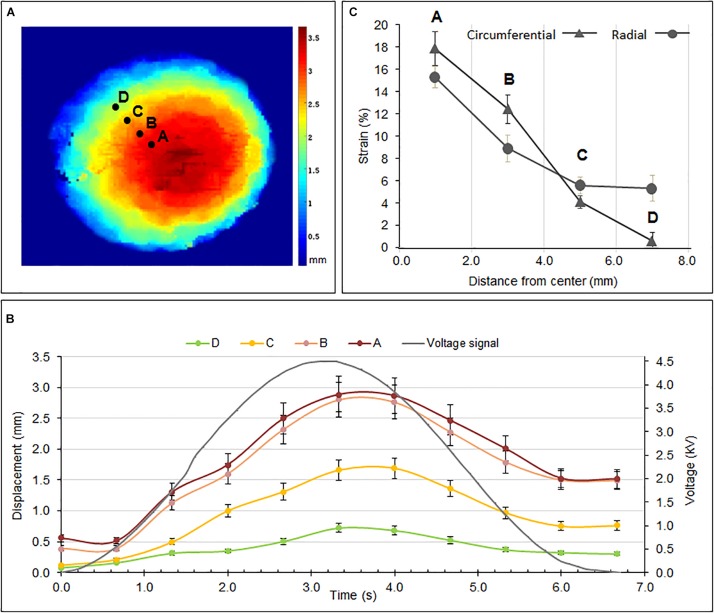
**(A)** Maximum displacement field map of the cell culture membrane for a sinusoidal voltage at 4.5 kV and 0.15 Hz; **(B)** displacement signals captured from the four points identified in **(A)** during one actuation cycle; **(C)** radial and circumferential strains estimated from the four markers shown in **(A)**. The unexpectedly large radial strain at point D may be due to locally loose constraints close to that edge, likely to have arisen from manufacturing defects. Error bars represent the standard deviation among three samples.

Specifically, the displacement field map was defined as the spatial distribution of the maximum vertical displacement (during one actuation cycle) of the cell culture membrane across its surface. It was determined using a 3D optical mapping system based on two stereo cameras (LI-OV580-Stereo, Leopard Imaging, United States) that captured images at regular time steps, when the bioreactor was at rest and under electrical actuation. Figure 3A presents the resulting map, showing that the highest displacements occurred, as expected, in the central region, reaching about 3 mm. Figure 3B presents the time evolution of the vertical displacement during one actuation cycle, as measured from the four markers identified in Figure 3A. The comparison with the co-plotted voltage signals shows that the displacements had a delay of about 0.5 s. This can be ascribed to a combination of losses derived from the viscosity of the elastomer materials used for the actuator’s and cell culture’s membranes, as well as the inertia of the coupling fluid between them.

In addition to the vertical displacement, the characterization included the radial and circumferential strains. Figure 3C presents their radial distribution, as estimated from the radial and circumferential displacements of the four markers identified in Figure 3C. The estimation was attained through 3D reconstructions by processing stereo images of the membrane at resting and deformed states.

As expected, both the strains were maximal in the central region of the membrane (about 18% for the circumferential strain and 15% for the radial strain) and decreased along the radial direction toward the edge. Nevertheless, it is worth stressing that this preliminary estimate of the strains was limited in accuracy, as it used only four markers and so the values could not be averaged over a large data set. This made the values particularly sensitive to defects inevitably introduced during the manual fabrication of the device, especially at the edges, where the membrane was likely constrained with anisotropic tension. This is evident, for instance, for the radial strain close to the edge (point D in Figure 3C), whose unexpected high value was probably due to a local loss of tension, possibly resulting in anomalous bumps.

### Mechanical Stimulation of Cell Cultures

In order to verify the bioreactor’s ability to mechanically induce changes on cells adhering to its surface, the following preliminary tests were conducted (all reagents were purchased from Sigma-Aldrich).

Fibroblasts from the HFFF2 (Human Fetal Foreskin Fibroblasts 2) line were cultured in supplemented 10% fetal bovine serum DMEM (Dulbecco’s modified eagle medium). Prior to cell seeding, the cell culture membrane was coated with type I collagen from rat tail. Fibroblasts were seeded at a density of 1,00,000 cells per bioreactor well and maintained in a cell culture incubator (100% humidity, 37°C, 5% CO_2_) for 24 h. Subsequently, the bioreactor was actuated inside the incubator with a unipolar sinusoidal voltage of 4.5 kV at 0.15 Hz. The stimulation lasted 8 h, which was considered sufficient to observe morphological changes in the cells, as it is known that fibroblasts subject to stretch begin to orient within 3 h (Neidlinger-Wilke et al., 2002).

The control sample consisted of an identical bioreactor, containing cells with the same passage number from the same cell batch, cultured in the same conditions, except for cyclic stretching (which was not performed, as no voltage was applied).

At the end of the test, the cells were fixed with 4% paraformaldehyde and stained for actin (phalloidin) and the nuclei (DAPI). The culture membrane was then imaged at different locations using a fluorescence microscope (IX81, Olympus, Japan).

**FIGURE 4:**
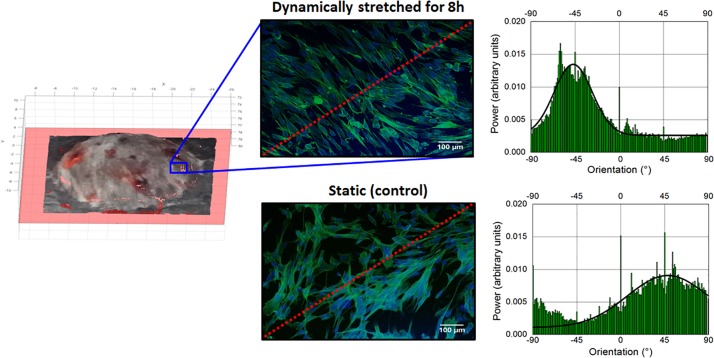
Effect of cyclic stretching of fibroblasts for 8 h in the bioreactor: images of a cell culture membrane’s patch (located between points C and D of Figure 3A), taken from both a dynamically stretched sample, just after stretching, **(top)** and the static sample of control **(bottom)**. The red dashed lines indicate the radial direction. The graphs next to each image present the angular distribution of the cytoskeletal fibers.

Figure 4 shows a typical outcome of this test, referring to a portion of the surface located between the points C and D identified in Figure 3A. Following the stimulation, the cytoskeletal fibers (stained in green) showed a distinct preferential orientation with respect to those of the control (non-stimulated) sample.

In order to quantify this effect, the green-stained fiber alignment was measured via a Fourier component analysis performed by the Directionality plug-in of the Fiji – ImageJ open-source software. The angular orientation of the fibers was computed with respect to a 0° reference defined as the horizontal right-hand direction of the image, with angles increasing counter-clockwise. The results are plotted next to each image in Figure 4: the cells in the stretched sample had a pronounced preferential orientation, with a higher intensity (peaking at −47°) and a narrower dispersion (±19°) than those of the control sample (peaking at 48° with a dispersion of ±41°).

This preliminary investigation suggests that the HC-DEA-based bioreactor is able to induce a measurable effect on the orientation of the cytoskeleton and that this orientation tends to be perpendicular to the direction of the imposed radial stretch.

A more in-depth assessment of this effect will require systematic testing on a large number of samples, with a diversity of conditions of stimulation, which goes beyond the scope of this Brief Research Report. Nevertheless, it is worth noting that this outcome is in accordance with previously reported findings. Indeed, using other stimulation devices, fibroblasts have been shown to align perpendicularly to the direction of uniaxial cyclic stretch (Huang et al., 2013; Weidenhamer and Tranquillo, 2013). Kang et al. (2011) suggested that when the actin filaments are cyclically stretched, a perpendicular alignment with respect to the direction of stretch emerges in response to nodal repositioning, to minimize net nodal forces from filament stress states. Similarly, other types of cells, such as lymphatic endothelial cells, have been reported to orient perpendicularly to a uniaxial 10% strain at 0.1 Hz applied for 24 h via a planar DEA-based device (Poulin et al., 2016).

Compared to previous studies, the new device proposed here offers a compact and versatile tool for applying strain fields that are not purely uniaxial, nor purely planar, without any additional mechanical equipment. This bioreactor could thus be used to investigate the response of cells stimulated by stretchable substrates undergoing out-of-plane deformations, thereby closer to several conditions *in vivo*.

### Future Developments

In addition to systematic testing, future developments should also optimize the actuation elastomer. In this study, the VHB acrylic by 3M was used to facilitate manufacturing, due to its adhesive properties, and take advantage of its high quasi-static electromechanical performance (Pelrine et al., 2000). Nevertheless, its high viscosity not only limits the driving frequency to the order of 1 Hz (which however is not a problem for a bioreactor, considering that cells *in vivo* are never exposed to much higher frequencies), but also can cause a significant stress relaxation, especially with the high pre-strains required for optimal operation (Pelrine et al., 2000). Therefore, to avoid a possible reduction of performance over time, less viscous elastomers, like silicones, are a better choice (Maffli et al., 2015; Rosset and Shea, 2016), enabling devices that should last million cycles (Matysek et al., 2011).

Furthermore, improved manufacturing is necessary to reduce manual procedures, which in this work inevitably determined the variability of performance evidenced by the error bars in Figures 2e,  3.

The main drawback of this technology is the need for high voltages (kV), although they have different implications, as discussed below, on the electrical driving units, the cultured cells, the operators and co-located electronics.

In terms of driving units, the generation of such voltages is not technically problematic, as the bioreactor does not require high powers and high frequencies. Indeed, the actuator is a capacitive load, which does not absorb high power, and the order of magnitude of frequencies needed for biomimetic cellular stretching is not greater than 1 Hz. For these reasons, in this work it was possible to use the high voltage multiplier by EMCO, which generates up to 5 kV at 0.5 W from an input signal up to 5 V, with a limited bandwidth (about 1 Hz) and a volume of about 2 cm^3^. Therefore, this kind of bioreactor can be controlled with battery-operated compact units.

In terms of interference with cell function, it is worth noting that the culture membrane is not exposed to the high electric field that builds up between the actuation electrodes. Indeed, the main field is confined within the actuation membrane, whilst the fringe field is not expected to impact the cell culture, due to the geometry of the device. In any case, it is useful to consider that previous studies on DEA-based cell stretchers, which have exposed cells to high fringe fields, have not obtained evidence of any effect on cellular (cardiomyocytes) viability (Imboden et al., 2019).

In terms of safety for the operators and for electronics which may be connected to the bioreactor (e.g., recording/stimulation electrodes and sensor probes), high voltages introduce the risk of electrostatic discharges (at a low power), which are unpleasant for humans and potentially destructive for electronics. This requires proper insulation of all the high voltage parts, including the voltage multiplier and the leads to the bioreactor. In this work, the high voltage unit was located outside the incubator and had thin cables arranged under the incubator’s closed door. As a future simplification, the compact unit could be sealed within the bioreactor’s case, obtaining a self-contained system, which could safely be used inside the incubator.

So, overall, there are no practical or safety concerns that should discourage the use of this technology just because of the high voltages. Nevertheless, they certainly are a drawback, not only because they bear the risk of electrostatic discharges (e.g., in case of breakdowns), but also because they make the electrical unit bulkier and more expensive than what it could be if the voltages were reduced by one order of magnitude. So, future developments should target a decrease of the voltages to a few hundred Volts. The critical threshold is around 250 V, which is related to low-size and low-cost drivers available for several piezoelectric transducers. To this end, efforts are needed to manufacture reliable DEA membranes with a thickness reduced to a few microns. Although this is challenging, the feasibility has already been proved (Poulin et al., 2015). However, as a lower thickness would reduce the membrane’s stiffness, it will be necessary to stack multiple thin dielectric layers intertwined to compliant electrodes, so as to enable both low voltages and adequate stiffness.

## Conclusion

We described a novel bioreactor to cyclically stretch cells *in vitro*, via electrically deformable elastomeric membranes with a dynamically tuneable curvature. As compared to state-of-the-art devices, this bioreactor avoids the need for external pneumatic/fluidic drivers or electrical motors, which typically make the whole system bulky and difficult to operate. The electrical tuneability of the membranes is advantageous to obtain compact, self-contained, lightweight and versatile devices.

The bioreactor has a modular structure with an interchangeable cell culture unit analogous to that of multi-well plates, enabling the use of standard cell assays.

We demonstrated that cyclic stretching for 8 h could induce significant changes in the directionality of fibroblast cytoskeletal fibers, encouraging systematic investigations of this new technology.

Although previous studies have already introduced dielectric elastomer actuation for cell stretching, the new configuration described here can generate strain fields that are not purely uniaxial, nor purely planar, but are instead based on the modulation of the cell culture membrane’s curvature. This feature opens up new opportunities to exploit this smart-material-based actuation technology to mimic complex 3D deformations occurring *in vivo*, such as those related to pulmonary inflation, cardiac and vascular pulsation and gastro-intestinal peristalsis.

## Data Availability Statement

All datasets generated for this study are included in the article/Supplementary Material.

## Author Contributions

FC, JC, and MG conceived the work. FC, JB, and AA supervised the work. JC and MG developed the bioreactor and performed the experiments. VM and VF measured the strains. FC and JC wrote the manuscript, with contributions from AA and JB.

## Conflict of Interest

The authors declare that the research was conducted in the absence of any commercial or financial relationships that could be construed as a potential conflict of interest.
